# “You Want to Catch the Biggest Thing Going in the Ocean”: A Qualitative Analysis of Intimate Partner Stalking

**DOI:** 10.1177/0886260520958632

**Published:** 2020-09-17

**Authors:** Caroline Flowers, Belinda Winder, Karen Slade

**Affiliations:** 1 Nottingham Trent University, United Kingdom

**Keywords:** intimate partner stalking, qualitative, interpretative phenomenological analysis (IPA), cognitive characteristics, stalking behaviour, perpetrator

## Abstract

This study employs a qualitative phenomenological exploration of the “lived” experiences of male intimate partner stalking (IPS) perpetrators serving a custodial sentence in the United Kingdom for an offense related to intimate partner violence (IPV). The purpose of this study is to capture the nature and complexity of the experiences of the pathway to IPS from the perspective of the perpetrator. The study seeks to provide a unique understanding of how IPS perpetrators attribute meaning to their behavior, illuminating the underpinning cognitive characteristics and emotions that play a role in their behavior. Semi-structured interviews were conducted with seven men with a history of IPS behavior. The resultant transcripts were analyzed using interpretative phenomenological analysis (IPA). Five superordinate themes reflecting participants’ experiences were identified: (a) “The quest for attention and affection creating connection,”(b) “Conflicted identity and extremes of self,” (c) “My life, a film set,” (d) “Gameplaying: One step ahead,” and (e) “Severed connections, changing the Gameplay.” The findings are presented in relation to the existing literature and theoretical frameworks which seek to explain stalking perpetration. The analysis provides a greater understanding of men who have engaged in IPS behavior, demonstrating how hearing the perspective of the perpetrator has value in informing theory and intervention. The study has provided valuable insight into the cognitive characteristics of this population and a rich understanding of the profiles of men who have engaged in IPS behavior. Implications for forensic practice, policy, and research are outlined, and recommendations for future research and overall limitations are discussed.

## Introduction

Intimate partner violence (IPV) and intimate partner stalking (IPS) behavior are two forms of interpersonal criminal behaviors increasingly coming to the attention of the criminal justice system (Melton, 2012). Stalking is a widespread criminal, social, and public health problem (Kropp et al., 2011), and a complex, distinct, and relatively new crime compared to other criminal behaviors (Brady & Nobles, 2017). Stalking encompasses a diverse range of behaviors from repeated and unwanted telephone calls, sending text messages/emails/letters, sending or leaving unwanted items, using social media, surveillance, loitering, proxy stalking, property damage/invasion, threats, and interfering with personal items (McEwan et al., 2012; McEwan et al., 2018). Stalking can escalate to sexual violence, physical violence, and homicide (McFarlane et al., 2002; Sheridan & Roberts, 2011).

Internationally, across a range of disciplines, defining and conceptualizing stalking has been the subject of debate (Fox et al., 2011). Whilst there is no universal agreed definition of stalking (Owens, 2016), most definitions capture a pattern of repetitive unwanted pursuit, a credible explicit or perceived threat, and the experience of fear by the victim (Logan & Walker, 2017; Miller, 2012; Spitzberg & Cupach, 2007). Stalking has been described as “psychological terrorism,” reflecting the psychological impact experienced by victims (Mullen et al., 2001). International studies indicate one in four to one in six individuals will become a victim (Mullen et al., 2009). The United Kingdom Office for National Statistics (2019)  indicates that approximately 2.5 million people experience stalking each year. Gender estimates vary across the literature. Whilst males and females engage in stalking behavior and both experience victimization (Spitzberg et al., 2010), most perpetrators are male, particularly in cases of IPS (Logan, 2010).

As the stalking research has evolved, theoretical models have been developed to explain stalking perpetration (Langhinrichsen-Rohling, 2012). Each explains stalking behavior in differing ways, attachment styles, gene selection, sociocultural influences, power and control, and patriarchy (Birch et al., 2018). The most widely acknowledged model is adult attachment theory (Patton et al., 2010), describing stalking as a behavioral expression of attachment pathology manifesting in relationship instability and problematic relationship styles. Alternatively, a psychodynamic perspective (Meloy, 1998) postulates that stalking emerges from a combination of personality characteristics (i.e., pathological narcissism) and attitudinal factors. In contrast, an evolutionary perspective (Duntley & Buss, 2012) suggests hunting is a basic human instinct, seeing the pursuit of women evolving from within-gender competition. Alternatively, relational goal pursuit theory (RGP; Cupach & Spitzberg, 2014) adopts a social psychological perspective to explain the obsessive nature of stalking, suggesting life goals represent positive outcomes, bringing higher order goals of self-worth. Blockages to these goals generate a cycle of negative emotional response, driving persistent pursuit. Whereas, Stark (2009) suggests IPS is a form of coercive control, driven by male dominance and a sense of ongoing entitlement to control a partner. Whilst these models provide a framework for understanding stalking perpetration, limited studies have empirically tested these theories (Nobles & Fox, 2013). Consequently, there is a lack of consensus explaining stalking perpetration (Spitzberg & Cupach, 2007).

### Intimate Partner Stalking: Key Issues for Consideration

Stalking perpetrators are not a homogenous group, presenting with different victim groups, psychopathology, clinical characteristics, and motivations (Mullen et al., 2009). The United Kingdom Office for National Statistics (2017)  indicates 54% of reported stalking offenses occurred within the context of IPV. Research suggests 45% of stalking cases occur following the dissolution of an intimate relationship (Spitzberg et al., 2010). IPS perpetrators form the largest stalking category (Logan et al., 2010; Mohandie et al., 2006; Spitzberg & Cupach, 2007). They are deemed the most persistent and dangerous (McEwan et al., 2009; Mullen et al., 1999), presenting with a higher risk of severe and lethal violence (James & Farnham, 2003; Rosenfeld, 2004; Sheridan & Roberts, 2011), compared with nonintimate partner stalkers. This group is more likely to use threats and violence compared to other subtypes, and is more likely to act with violence following threats (McEwan et al., 2012; McEwan et al., 2017). On average two women are killed in England and Wales by a current or former partner each week (Home Office, 2010; Women’s Aid, 2018). These figures are supported by research, indicating a link between IPV, stalking behavior, and homicide (Monckton-Smith et al., 2017). IPS perpetrators are likely to continue to pursue victims following legal deterrents (Logan & Walker, 2009; Mohandie et al., 2006). Due to the prior relationship history, IPS perpetrators possess background knowledge on the victim, enabling them to facilitate stalking behavior (Logan & Walker, 2009).

The existing literature suggests a link between IPV and stalking behavior (Douglas & Dutton, 2001; Gerbrandij et al., 2018; Logan, 2010; McEwan et al., 2017). Nonetheless, the underpinning factors which may play a role in this connection are yet to be clarified. A key debate focuses on whether IPS should be conceptualized as a variant or continuation of IPV, or a distinct but related offense. Commonly, IPS has been conceptualized as a continuation of a pattern of male-perpetrated partner violence and coercive control which persists against a partner following the breakdown of a relationship (Douglas & Dutton, 2001; Logan & Cole, 2011; Logan & Walker, 2009; Norris et al., 2011; Stark, 2009). It is argued that this broad conceptualization assumes a gendered view of IPV (McEwan et al., 2017), adopting the premise that IPV during a relationship and post-relationship stalking are the same concepts. A critical debate focuses on the challenges of separating what patterns of behaviors distinguish IPV and stalking behaviors as both present similar behaviors (i.e., psychological abuse, threats, physical violence, monitoring, and surveillance). Consequently, McEwan et al. (2017) propose a narrower conceptualization which differentiates between IPV and stalking. Under this conceptualization, in the context of a current intact relationship any form of psychological or violent behavior should be defined as IPV, whereas following the dissolution of the relationship any form of psychological or violent behaviour should be defined as stalking and termed post-relationship stalking. Indeed, in the United Kingdom, the stalking legislation in England and Wales (Protection of Freedoms Act, 2012) adopts this definition, failing to define stalking as occurring when the relationship is intact, employing the term “coercive control” to criminalize stalking behavior when the relationship is intact. Nonetheless, existing literature suggests that stalking behavior can occur both while the relationship is intact and following the dissolution of the relationship (Cox & Speziale, 2009; Ferreira & Matos, 2013).

Extensive research has explored IPV perpetrators (Capaldi et al., 2012). In comparison, less focus has been given to IPS perpetrators. One area for consideration is the gaps in knowledge base for IPS. Research to date has focused on victim and perpetrator samples, employing retrospective observational study designs with data obtained from case files, police records, psychometrics, and surveys (Nijdam-Jones et al., 2018). No qualitative studies have explored the characteristics of IPS from the perspective of the perpetrator nor have studies been conducted in prison settings. Whilst cognitive characteristics (i.e., schemas or “implicit” theories) have been explored in other offense types (Beech et al., 2005; Polaschek & Gannon, 2004) and IPV perpetrators (Gilchrist, 2009; Weldon & Gilchrist, 2012), research exploring cognitive characteristics, and particularly the role of obsession and fixation, remains unexplored in IPS perpetrators. Both these terms are used interchangeably within the literature. Obsessive thinking captures the perpetrator’s preoccupation and unusual interest with the victim, devoting excessive periods of time talking or thinking about the victim (Kropp et al., 2008; Meloy & Fisher, 2005). Whereas fixation is defined as an intrusive and intense preoccupation with another person or activity. Whilst individuals experience “normal” fixations in everyday life, in the context of stalking behavior, these are classified as pathological fixations which are obsessive preoccupations (MacKenzie et al., 2009). Nonetheless, whilst obsession and fixation are assumed to be a key factor underpinning and drive stalking behavior, the function of this are yet to be empirically tested (Birch et al., 2018).

## The Present Study

This article focuses specifically on male IPS. For the purpose of this article, the term “intimate partner stalking” depicts an individual who has engaged in stalking behavior towards a female romantic intimate partner at any stage in the relationship history. Thus, capturing stalking behavior both when the relationship was intact and following the dissolution of the relationship. This definition seeks to capture current or former partners, whether legally married, separated, divorced, cohabitants, relationships classified as dating in the context of girlfriend/boyfriend, and other types of intimate partners such as extramarital affairs or multiple relationships.

The current study seeks to explore IPS through the eyes of the perpetrator using a phenomenological approach to capture the “voice,” experiences, and cognitions of men who have engaged in IPS. The study aimed to address the following research questions: (a) What personal meaning do men who have engaged in IPS attribute to their stalking behavior and (b) What are the cognitive characteristics that underpin the pathway to IPS? The primary aim is to obtain insight into the experiences and personal meaning perpetrators of IPS attribute to their stalking behavior and experiences of the stalking pathway. For the purpose of this study, the term “pathway to IPS stalking” intends to depict the experiences during the prestalking period (i.e., initially meeting the victim), the relationship history (i.e., when the relationship was intact), and post relationship with the victim, thus capturing the wider context of the stalking episode including stalking perpetration culminating in violence. This approach seeks to illuminate the triggers, emotions, and cognitive characteristics underpinning stalking behavior and stalking violence.

Research exploring cognitive characteristics are valuable in assisting the development of formulation models and informing criminogenic needs which can be targeted through intervention (Ward, 2000). Thus, the findings are intended to inform approaches to the clinical management and intervention for IPS perpetrators. This study utilizes interpretative phenomenological analysis (IPA) to capture individuals’ experiences and how they make sense of their personal and social world (Smith et al., 2012). IPA studies are expanding into the field of forensic psychology (Murphy & Winder, 2016; Nulty et al., 2019), enabling focus to be given to complex unexplored issues, and diverse and hidden groups within society. Thus, a qualitative approach utilizing IPA imparts a voice to IPS perpetrators (Waldram, 2007).

## Method

### Selection Criteria

Seven male participants serving a custodial sentence for an offense related to IPV formed the sample. Participants were recruited from four separate prisons in the United Kingdom. Participants met the following inclusion criteria: A history of IPV (i.e., conviction, police callouts, self-reported) and evidence of self-reported or a conviction for stalking or a stalking-related offense such as harassment and breaches of supervision orders or campaigns of harassment exceeding the two-week threshold outlined by Purcell et al. (2004) against either a former or current partner. Level of responsibility taking and completion of intervention were not exhaustive exclusion/inclusion criterion.

### Sample

IPA adopts a purposive sampling approach. The sample size of seven was deemed sufficient to explore similarities and differences between cases, and thus appropriate for IPA methodology (Smith et al., 2012). Table 1 presents key information pertaining to participants.

**Table 1.** Participants Demographics and Offense Details

**Participant Number**	**Index offence Note: *All participants had been convicted of an offence related to IPV not stalking***	**Prior known history of IPV and supervision violations towards the stalking victim**	**Relationship to victim at time of index offence**	**Another relationship (not with the stalking victim) at the time of escalation in stalking behaviour/stalking violence**	**History of stalking another partner**	**Employed**
1	False Imprisonment, Threats to Kill, Rape, Attempted Murder	Yes Issued with harassment order	Former partner	Yes – Entered into new live in relationship	Yes – 2 victims	Yes
2	Sexual assault	Yes Breached bail	Former partner	Yes Was in a ‘live in relationship’ with two partners – one of which was stalking victim	No – 1 victim	No
3	Attempted murder	Yes Breached restraining order Harassment offence	Former partner	First victim work colleague. Self-reports intimate relationship. Married at time of stalking campaign Second victim (unknown)	Yes – 2 victims	Yes
4	Wounding and other acts endangering life	No Breached restraining order	Former partner	No	No – 1 victim	No
5	Rape (partner) Harassment & Affray	Yes Breached restraining order	Former partner	Yes – Entered into new live in relationship	No – 1 victim	No
6	Sexual assault on female	Yes Breached restraining order Harassment	Former partner	Yes – Entered into new relationship	No – 1 victim	No
7	Murder	Yes On bail at time of murder	Former partner	Was in a ‘live in relationship’ with two partners one of which was stalking victim. Married to another partner and had ongoing relationship with a third woman.	No – 1 victim	Yes

All participants were classified as white British, with an age range of 26 to 58 years at the time of the study. No participant had a conviction for stalking. Three had a conviction for harassment. All self-reported engaging in stalking behavior, culminating in an act of physical and/or sexual violence, with one committing lethal violence. All had breached supervision orders. Six had a prior known history of IPV. Six were in a relationship with another partner at the time of the stalking campaign. None of the participants denied the IPV index offense or the stalking behavior. Six participants had completed the Healthy Relationships Program, a high intensity cognitive-behavioral intervention designed to target the criminogenic needs of men who have a history of IPV offending.

### Recruitment

Ethical approval was obtained from the National Offender Management Service (NOMS) and a UK university. Based on practicalities, potential participants were identified by a prison-based gatekeeper. Those meeting the inclusion criteria were sent a letter and background information sheet. The first author met with participants privately to discuss participation. Full informed consent was obtained. No participants withdrew consent.

### Procedure

Semi-structured, one-to-one interviews are deemed appropriate methods to generate IPA data as they facilitate rich narrative accounts of participants’ experiences (Smith et al., 2012). A semi-structured interview schedule was designed, providing a flexible data collection tool to capture the psychological/social worlds of participants. This focused on broad areas of relationship history with the victim, response to relationship breakdown and life problems, and reflections on stalking behavior and stalking violence. A pilot interview was conducted involving one participant, whose datum was included in the study. All participants were interviewed separately by the first author. Interviews were conducted in private prison interview rooms, lasting between 55 minutes and 3 hours (Mean: 2 hours 18 minutes), and were recorded using an audiotape recorder. Participants were encouraged to tell their stories of the pathway to their stalking behavior in their own words. Consistent with IPA methodology, the interview schedule was used as a guide and prompt, which did not dictate the direction of the interview, but rather encouraged the “discovery” of participants’ experience. Participants were encouraged to describe their experiences in a way which was meaningful to them, achieved by being responsive to their individual needs. Participants were provided with stimulus material in the form of a visual time line presented on a flip chart to act as a prompt to narrate the pathway to IPS. This proved useful for participants as they chose to tell their stories as a chronological narrative of their pathway and key events relevant to their experience. All participants told their stories their own way with the researcher interjecting with open questions to prompt and encourage reflection (i.e., “If you were looking in now and winding time back, what kind of person would we see? What were you saying to yourself at the time when you did that? Tell me why you wanted revenge?”).

### Data Analysis

Transcripts were analyzed within a qualitative framework according to the principles of IPA (Smith et al., 2012). IPA was deemed a suitable approach as the study seeks to elicit understanding of IPS experiences and how they attribute meaning to their pathway to stalking behavior. The central assumption of IPA is that participants are experts in their own lives (Smith et al., 2012). IPA employs “double hermeneutics” whereby “the participants are trying to make sense of their world; the researcher is trying to make sense of the participants’ trying to make sense of their world” (Smith & Osborn, 2003, p. 51). This idiographic approach allows for a flexible and rich insight into participants’ psychological world, thereby providing an insightful exploration of participant’s sense of self, cognitions, motivations, and feelings underpinning their experiences. The data were prepared in line with Smith et al. (2012) transcription guidelines. Interviews were transcribed in full by typing out the dialog verbatim and anonymized. Language, grammar, and words of participants were not modified. Analysis was inductive and developed iteratively through a series of stages to identify patterns of meaning (i.e., themes) in the data. Analysis adopted a case-by-case, in-depth analysis of each transcript separately followed by an integration of cases. Analysis comprised of several stages: Reading and rereading of transcripts, notetaking, development of emergent themes, searching for connections across emergent themes, and grouping them together across accounts. When the first case was saturated, the next transcript was analyzed. Due to the iterative nature of IPA, analysis required constant reflection and reexamination of the transcripts and themes. A summary table of superordinate and subordinate themes was constructed for each participant. The final stage of analysis involved comparing themes across cases to consider the interrelationship to explore similarities and differences between participants. A master table of themes was produced outlining all superordinate and subordinate themes across participants.

### Reliability, Validity and Reflexivity

Qualitative research has historically been subject to criticism (Braun & Clarke, 2013). Consequently, frameworks for conducting high standard qualitative research were developed (Braun & Clarke, 2013; Yardley, 2000). In keeping with the recommendations made by Smith et al. (2012), throughout the research process measures were implemented to ensure the validity and reliability of the research. This was achieved by adopting Yardley’s evaluative criteria, focusing on four key principles of sensitivity to context, commitment and rigor, coherence and transparency, and impact and importance. Sensitivity to context was demonstrated throughout the early stages of the research process—initially through demonstrating an appreciation of the existing literature and gaps in understanding IPS perpetrators, through the rationale to give voice to this complex group, and the rich dataset obtained through the engagement and approach employed to interviewing participants. The verbatim extracts ensure the article meets the aims by giving a voice to participants and grounding arguments in existing literature. The second principle, commitment and rigor, is evident through the personal commitment of the authors. Interview and analysis took a significant period of time, with the researcher concluding that the data had reached saturation (Saunders et al., 2018). Themes were consistently checked against the transcripts to ensure they were grounded in the data and were representative of participants’ accounts. Analysis moved beyond description and was interpretative, thus presenting key information regarding participants’ collective experiences. Each stage of analysis was reviewed by the second author, thereby acting as an independent audit. The third principle transparency and coherence has been achieved through clarity regarding participant selection, interview process, analysis, and write-up of the article. Through adopting a novel methodology, the article provides a valuable contribution to the stalking literature, thus meeting the final principle of impact and importance. The authors recognize reflexivity is central to a qualitative method, providing transparency with the research process. The lead author brings to this article a breadth of experience in historically working with men who have committed IPV offenses, enabling the integration of forensic practice and research. Whilst it is recognized this may influence interpretation of the data, this is considered a strength of the study, facilitating the exploration of a sensitive subject area to encourage and access participants’ experience, evident by the richness of the data obtained.

## Results

IPA analysis identified five superordinate themes: “The quest for attention and affection creating connection,” “Conflicted identity and extremes of self,” “My life, a film set,” “Gameplaying: One step ahead,” and “Severed connections, changing the gameplay.” The superordinate themes are interlinked and presented in turn to reflect the representation of the pathway to IPS and the escalation to stalking violence from the perspective of the perpetrator. The superordinate themes were salient in the accounts and experiences of all participants, with a varying degree of similarity and divergence of each subordinate themes within each individual narrative (See Table 2).

**Table 2.** Superordinate and Subordinate Themes

**Theme Number**	**Superordinate Theme**	**Subordinate themes**
1.	The quest for attention and affection creating connection	The thrill of the chase: ‘Proving you can get a partner is like a drug’ Obsessive desires: ‘I know I was obsessed with her’
2.	Conflicted identity and extremes of self	Portraying the ideal self to the world: ‘I will be successful I won’t be beat’ Saying one thing, doing another: ‘It was like a tug of war’ Life has gone off script: ‘It was a cocktail of little things’
3.	My life, a film set	Exaggerated perspective – recounting the script She went off script – mixed messages
4.	Gameplaying: ‘One step ahead’	Knowledge is power: ‘I knew where she was on day-to-day basis’ Desire to win: ‘A battle of wills and I was winning’
5.	Severed connections, changing the gameplay	Spiralling emotions: ‘In one of those snowdomes going around in circles’ Restoring pride and elevating the self: ‘A red rag to a bull’

### Superordinate Theme 1: The Quest for Attention and Affection Creating Connection

This salient theme encapsulates how participants articulated a narrative of seeking affection and attention in pursuit of a connection. This plays out in several stages of relationship development: the initial attraction phase and relationship pursuit, during the relationship, and after relationship dissolution . A strikingly unique finding encompasses how six participants described complex relationship dynamics. Negotiating these relationship dynamics were the catalyst for stalking behavior. A second element captures participants’ experiences of fantasy and obsession. Whilst there were some variations in how this was experienced by participants, there were similarities in how this manifested in the subordinate themes.

#### The thrill of the chase: “Proving you can get a partner is like a drug.”

This subordinate theme captures how participants described craving affection and attention. This follows two intertwined pathways: seeking a connection and bringing the highs of pursuing a new relationship. It also encapsulates the desire to pursue following relationship dissolution. Throughout participants’ narratives they use strong descriptive words such as “buzz,” “challenge,” “work at it,” “within your power,” and “satisfaction,” suggesting that pursuit brought excitement, a sense of adventure, and something to be attained. There was a sense that participants’ accounts portrayed a sense of relishing in the attention and affection received from pursuing connections. This theme is echoed in the “storytelling” process of a participant, one where he portrays his experience as a chase and a desire for affection and love beginning in the lust and attraction stage of the relationship, through to stalking perpetration:

I did start seeing someone else … someone who showed me lots of affection … I enjoy that … but maybe I need to look into why you are giving me affection sometimes rather than just letting me get involved in a relationship with someone I don’t really want to be with … it was a buzz, it was like being a naughty boy getting his wicked way … (P1, 309–313) … It’s wanting to be accepted when you are not accepted and wanting the unacceptable … knowing you can achieve them little goals is like a drug to do things, in the end it’s something you don’t really want but you end up going down a wild path … so you only want to catch a stickleback and you catch it, and then suddenly you want to catch a pike, and then you want to catch a whale, and then you want to catch the biggest thing going in the ocean. So, it’s sort of like a little chase … you just want love at the end of the day … and then because she don’t want me no more I want her so I will try and get her …it’s like let’s prove what I can achieve (P1, 585–608).

At a hermeneutic level, his use of metaphors appears to represent the underpinning belief that if a male is patient, he will obtain the object of his desires. There is the underpinning assumption that someone is better than nobody. Participants articulated a narrative portraying what they were getting out of the relationship (i.e., attention, affection, a home, employment, and financial gain) rather than what they were giving. As participants told their stories and reflected on their experiences of relationships, they shared a common theme—complex relationship dynamics. This is exemplified in the following extracts:

I got into that one (relationship with second victim) before that one had properly finished (P1, 317); I was with two women at the same time so it’s complicated (P2, 13); I think that I should also mention that I was married at the time … (P3, 12–15); I sort of met someone else (P5, 313); So I have gone up to my friends and having a party and having a one-night stand (P6, 62–62); The relationship I had with the deceased, my victim, erm she wasn’t … I was married at the time to another woman …There is also another woman in this (a third partner; P7, 165–166).

In pursuit of connections, participants described complex relationship dynamics, pursuing new connections, one-night stands, multiple relationships, double lives, or overlapping relationships. Whilst all participants described engaging in stalking behavior during the relationship with the victim, the stalking behavior escalated following the dissolution of the relationship. It is noteworthy that the victims were not the partners they regarded their primary relationship at the time of the escalation in stalking tactics and violence. The majority of participants had either entered into a new relationship, were married to another partner, or for those living double lives continued the relationship with the second partner. That is, participants continued to stalk their former partner following the dissolution of the relationship whilst in a relationship with another partner. At a hermeneutic level, there was a sense that within the pathway to IPS, participants idealized the victim, or rather idealized what the victim represented in his life.

#### Obsessive desires: “I know that I was obsessed with her.”

This subordinate theme captures the most prominent element of the theme echoed by all participants. It represents the experiences of fantasy and obsessive love which becomes all-encompassing, playing out through all stages of the relationship history and stalking perpetration. The collective narratives initially portray a “Romeo and Juliet” type love story, whereby participants talked of instant attraction and infatuation, with thoughts of eternal togetherness and savior like qualities. Underpinning the collective narrative was an element of a fantasy and striving for a perfect relationship. Participants’ use of language throughout the storytelling process is peppered with powerful metaphors and gestures of an everlasting bond. This is exemplified in the following extracts:

I have had other relationships … the feeling was totally different … something connected into my heart (P5, 11–16). I know that I was obsessed with her … because I didn’t want to let go, and I thought she was mine … no one else is having her and that’s obsessed, I was obsessed with her (P5, 210–213).

With her it was like a light bulb as there was nothing that I had felt that instant attraction to somebody (P3, 541–542). With the first one (victim) there came a point very quickly where there was an attachment …I loved her, that’s when it sort of struck me for the first time I think I grew to love my wife … (P3, 125–131). I followed the car at a distance sort of shadowing stalking her … It was a case of just wanting to be near her … wanting to know what she was doing, who she was with and it became all-encompassing to the stage where I virtually wanted to know everything about her life in a sense erm even by just driving up and seeing if they were in the house and if the cars were there and then driving away (P3, 159–167).

I loved her from day one, from a teenager … I just wanted to be with her (P2, 158–170). I couldn’t imagine life without her (P4, 120). She meant everything to me … I did want it to be forever, my world (P6, 162).

At a hermeneutic level, participants’ narratives and the use of metaphors signifies the strength of their obsession which was present at all stages of the pathway to IPS. There is a sense of overwhelming dependence underpinning their collective narratives, with rejection perceived as a temporary hold up, intensifying the desire for connection. It is noteworthy that participants sought to explain the desire for contact as addiction, in that they conceptualized how every form of contact there was a payoff, feeding the desire to continue the pursuit. The aforementioned extracts encapsulate the sense that obtaining any form of contact brought fleeting satisfaction, following which the cycle resumed.

### Superordinate Theme 2: Conflicted Identity and Extremes of Self

This theme stems from the way in which participants portrayed how they presented themselves to the outside world, struggles with the persona they desired, and an inability to integrate aspirations and expectations of the self during the relationship. Central to this theme is a prior history of psychological violence, and in some cases physical violence, within the relationship history with the victim. In response to life problems, relationship dynamics and breakdown, participants expressed becoming more conflicted and uncertain. Within the collective narratives there was a sense of considerable internal cognitive tension on recognizing they are not living up to their personal standards. A striking element of this theme is a sense of ambivalence towards the victim, seeing love turn to hate.

#### Portraying the ideal self to the world: “I will be successful I won’t be beat.”

One distinct theme within the collective narratives was the sense that participants viewed the self at extremes: the self as powerful or powerless, winning or losing, successful or a failure, in control or out of control throughout all stages of the pathway to IPS. Underpinning the theme is a fragile sense of self. There is a sense of a “Walter Mitty” type character, whereby participants articulated a narrative of a successful self, which was portrayed to the world. There are strong expectations linked to identity and the role of “a man,” with narratives interspersed with the view of self as “the supporter,” “hero,” and “rescuer.” As participants told their stories, they conceptualized how the desire for status and success was a key factor in how they viewed themselves and wanted the world to view them. Whilst this theme was salient in the narratives of all participants, it was particularly striking within the narrative of participant seven:

I always needed to be seen as successful … it was materialist things it was all about fast cars and things like that … status was paramount. As I reflect back, I was clearly attempting to portray someone successful. I felt I needed to succeed in life (P7, 165–166).

Within his narrative he talked of using social media to present a persona of someone who was successful. Reflecting on his experience, he pinpointed underpinning this was a fear of failure, a theme which resonates powerfully throughout all the participants’ narratives. This theme is also strongly exemplified in the extract by participant four, who spoke of experiencing debilitating illness which impacted on his sense of self and identity. Similar to other participants’ experiences, his account portrayed how a life-changing event eroded his successful self, leaving a shell of a man with the loss of a positive social identity:

We had lived life to the full we had a very healthy love life … I had created the life for her, I gave her the opportunity, I had the financial power in them days when we first got together. She was still married. We had an affair. (P4, 102–108).

Through the collective narratives, participants spoke of experiencing similar setbacks in life (i.e., physical illness, depression, work, and relationship problems). From a hermeneutic level, their accounts describe problems with coping. Significantly, participants articulated a narrative which placed status and esteem as critical to the self, and when this was threatened, eroded the sense of self.

#### Saying one thing, doing another: “It was like a tug of war.”

Underpinning the narratives of all participants was the sense of personal inconsistency and contradiction between what participants said and desired, and the reality of their behaviors during the relationship and throughout the pathway to IPS. This incoherence between the two selves paved the way to a cycle of negative emotions and behaviors, subsequently resulting in their goal being pushed further away, that is, the very thing participants were striving to keep hold of, their victim. Within the experiences of participants, their accounts were peppered with references to perpetrating psychological and/or physical violence prior to the stalking behavior and stalking violence, which was at odds with their specific views about how men should behave under specific circumstances and within a relationship. Participants reflected on the contradictions of their desires to be a loving supportive partner and the reality of how their relationship had been entwined within a backdrop of psychological or physical abuse. This theme is exemplified in the following extracts:

I was shouting … nasty things name calling …. Don’t speak to me like that you slut … every name I could think of I would say it (P2, 45–47).

When her dad met me …they had said was look after my little baby girl and I always made that promise … Obviously I just wanted to be loved … I wanted to show the love back … (P5, 48–49). I admitted that I had pushed her I won’t deny that, I had probably slapped her … Of course, I don’t want to keep on having to get my hands on her … I am supposed to be protecting her I am supposed to be loving her I supposed to be showing her family that I care about her not having to slap her … I used to push the blame to her, and it wasn’t her it was me … (P5, 148–164).

When things went wrong, they went wrong … there was one argument … I just blanked out and when I come round I found my hands were wrapped round her throat … I didn’t want to treat her rotten, I always wanted to be there to support her as a partner and a family erm so I never wanted to do bad by her really …(P6, 31–49)

A striking feature in the narrative of participant five is the use of the repeated phrase “I am supposed to be,” highlighting the flux he experienced. Indeed, the contradictory self was prominent for all participants as they reflected on how this played out in their relationship and the pathway to IPS. Within this dissonance, there is hypocrisy, as they strive to be one thing, but this is inconsistent with their personal standards. At a hermeneutic level, narratives reflect the cycle of violence characterized by IPV (Walker, 1989), building from unexpressed anger and unresolved conflict, culminating in psychological and physical violence.

#### Life has gone off script: “It was a cocktail of little things.”

This theme stems from the perception that life is either in control or out of control during the relationship and after relationship dissolution. Whilst the way in which this unfolded for participants played out in different ways, common features related to struggles coping with relationship dynamics and the radiating impact on other areas of their lives. Significantly, a theme of loss appeared to underpin this, whether that be loss of a relationship, employment, and stability or loss of identity and status when the true self was exposed. Within the narratives there was a collective sense of putting on a mask to the outside world. Some participants spoke of a history of substance misuse, either drugs, prescription drugs or alcohol to cope. Participants described underpinning anxiety and self-loathing, becoming trapped in a cycle of self-hate which was projected onto victims. This theme is exemplified in the extract by participant one, who reflected on what the camera would have observed looking back in time:

I was taking the tablets, drinking more … not working when I would be normally …. working to cover up my emotions to escape … and just really taking more of these co-codamol tablets, so it was a little cocktail of things (183-18). We would see a character who was a scared person within himself … I hid it well (176-181). My life was in a disaster state … I think it had gone past the point of really caring and then its I want to be with her, no I don’t want to be with her ….it was something I didn’t know how to cope with (P1, 486–494).

For participant three, four and seven, there was a sense of feeling backed into a corner. Participant seven struggled to negotiate multiple relationships, seeing him become more controlling. This paved the way for what he describes as a cycle of psychological and physical abuse prior to homicide. The following extract illustrates the sequence this played in the pathway to stalking violence:

When I realized that the net was closing in a little bit of panic stepped in … I am going to have to watch what I put on Facebook … I liked the fact that she wanted to be in a relationship with me, but I didn’t want it to be fully exclusive … so I started putting conditions on the relationship … (P7, 278–284).

### Superordinate Theme 3: My Life, a Film Set

This superordinate theme represents how participants told their story, reflecting back on their relationship history and pathway to IPS, and how they recaptured experiencing their worlds. There is an overwhelming sense that participants portrayed themselves as a detached observer watching a play. It is notable that participants recounted the pathway in significant detail like reading the script of a film set and reliving part of the scene. Underpinning this theme are the cognitive characteristics that play a role in the pathway to IPS and significantly participants’ perceptions of the role of the victim. Participants talk of experiencing “mixed messages,” which from a hermeneutic level represents distortions of reality. A central feature which resonated through participants’ narratives was the view that no one was listening to their side of the story.

#### Exaggerated perspective: Recounting the script.

This theme stems from the way in which participants recounted their script. In the “storytelling process,” there is a sense that participants’ cognitive processes were exaggerated, with the perception they were under a microscope with the whole world looking in, with any flaw in their character being magnified and exposed for all to see. It is striking that participants’ accounts are highly interspersed with the pronoun “I,” with focus given to the ins and outs of “I did this” and “she did this,” suddenly fast forwarding to a highly pertinent point, reflecting on relationship breakdown, an act of vengeance or violence. From a hermeneutic level, there is a sense that his inner speech and inner voice are self-centered. It is like he is ruminating out loud with no sense of reality and a frantic thought process with ruminating monolog, and distinct lack of emotion and perspective taking.

#### She went off script: Mixed messages.

This theme conceptualizes how participants makes sense of their actions and pursuit of the victim during the pathway to IPS, and in doing so how they seek to protect their sense of self as not fundamentally bad. Crucially, the theme focuses on participants’ perceptions of the role of the victim and the view that participants perceived the victim as an actress in their play. Whilst this theme was evident in the narratives of all participants, the way in which this played out was different across cases. Participants one, two, three and five consistently made references to perceiving “mixed signals” from victims and experiences of ongoing contact with the victim. Whereas within the narratives of participants four, six and seven, focus was given to the role the victim played. As the following extracts suggest, from a hermeneutic level, there is a sense that participants believed what they want to believe. They looked at the relationship through one lens, failing to consider that the victim may in fact be fearful or attempting to appease or let them down gently. The following extracts powerfully encapsulate the narratives of all participants:

I was getting mixed messages from her …now and again …I would get a little text … she would tell me that she did love me and a couple of days later, I hate you, I don’t want you. So, I was getting a lot of mixed signals off her … does she love me, or does she hate me? Why is she telling me all these different things, that didn’t make sense to me, so I was thinking whether I am coming or am I am going, what is going on? … I said I need a final answer, but before that would happen we would start meeting again, sleeping together, doing everything we normally would …(P5, 212–224).

She would text me and say meet me here, so I went and said to her will I get done for breach of bail? She said its not me getting you done for breach of bail its my X interfering …. So I got done for these letters and these cards and now you are here sleeping with me … and that’s what happened. I couldn’t get my head round it its like you are meeting me here and I get done for sending letters … (P2, 85–99).

### Superordinate Theme 4: Gameplaying—“One Step Ahead”

This superordinate theme captures how participants depict their stalking behavior, during the relationship and after relationship dissolution, as gameplaying within the “storytelling” process. Within the collective narratives there is a sense that participants desired control over others and their environment. The theme takes two interlinked directions. Firstly, the subordinate theme “Knowledge is power: ‘I knew where she was on a day-to-day basis’” stems from the need for participants to attain knowledge of their victims and the methods employed to facilitate this. Secondly, the subordinate theme “Desire to win: ‘A battle of wills and I was winning’” captures the extremes of thinking and behavior and the striking need for control.

#### Knowledge is power: “I knew where she was on a day-to-day basis.”

One clear theme within all participants’ narratives was the need to attain knowledge of the victim and the tactics used. Participants expressed how obtaining knowledge gave power to be used against the victim. This begins in the relationship, plays out during the formation of the relationship, during the relationship, and after relationship dissolution. Strikingly, participants described the need to control the ending of the relationship. The following extracts powerfully encapsulate the narratives of participants:

I went to have a duplicate key cut… An opportunity was there, and why did I do that, I think it was about feeling involved in her life, not just learning more about her by basically sort of trespass …it gave me access back to the house when she wasn’t there so it allowed me to sort of trespass when I wanted to and I did … to actually delve into her personal life without her knowledge …it is an ultimate level of control … I had an element of control over her she wasn’t aware of …. (P3, 207/673–679). So I started to question what is going on … why do I need to know, it’s a control issue its about even though I am not actually in the relationship I have got no need at any point to know what somebody is doing in that sense, within a relationship, but this is not a relationship its finished its over, why do I need to know, but I wanted to know, its knowledge … (P3, 835–838).

Similarly, participants four and six reflected on how they had a strong desire to obtain information in response to quest for answers. Like participant seven, they used technology to monitor and later track their partners during the relationship and following the breakdown which gave them control over all aspects of their partners’ lives :

I set up an excel file on the phone where I could sift and prioritized what numbers had been called … I wanted to know how much, there was far too much information to go through, I just wanted to make it easier (P4, 192–204).

I had one of those (recorders), I used to leave the recorder running under the sofa when I was out so that I could go back to the recording and listen to it to find out what she had been doing that day. (P6, 366–369).

The following extract from participant seven illustrates the development of this pathway, beginning in the initial attraction stages by obtaining knowledge from social media as he pursued the relationship, stalking behavior within the relationship, and finally the pathway to stalking violence using social media and his interactions with the police to elicit information. The pathway to stalking and violence is set and is captured in the following extract:

The texts were sent to sublimely check that the mood was relaxed and not tense. It to me was a type of investigating tool on the status of the relationship. My monitoring was a way of possibly predicting further deterioration, identifying lies, a way of being one step ahead. My insecurities were fueling my desire to check up on her … if it was to end at least I could possibly have some dignity as a reason in case anyone asked. Engaging in these behaviors was certainly a way of me thinking I was in control … Was I doing it to calm myself? I believe it was about power … because knowledge is power. Knowledge from being or trying to be one step ahead … Part of my bail conditions were not to go to the house … the police rang me up and said we are aware that she has got to send you photos but don’t think this is an opportunity for you to contact her … I will never forget what he said. He goes she is moving on with her life she is going back to work (DING) and she has been through all this and she is moving on. So, I was like alright …I kind of thought to myself I need to go … I went up the night on the X and she was murdered on X (P7, 506–515).

Throughout his narrative, he likens Facebook to an “investigating tool” which from a hermeneutic level is portrayed as a covert method of investigation whereby he secretly gathers information. He portrays how knowledge brings power, relief, and answers, and the ultimate level of control. In the pathway to homicide there is a sense that he is piecing together the jigsaw to assist in his plan for revenge.

#### Desire to win: “A battle of wills and I was winning.”

This subordinate theme reflects the collective narratives of all participants. The language participants used is reflective of gameplaying; “a battle,” “the winner’s position,” “winning,” “losing,” “who has the control,” “shifts in control” and the “buzz,” “satisfaction and challenge of winning,” and “game changer.” There is a sense that the victim is on a yoyo with participants casting the victim out and reeling her back in to meet their needs. This begins with psychological violence within the relationship and plays out in the stalking episode both during the relationship and after relationship dissolution. There is a sense of willful dominance which becomes a conquest; a game where they are seen to be winning or losing. The concept of control resonated throughout all the participants’ narratives. In the storytelling process there was a sense that the desire to win heightened self-esteem from their perception of having control. The following extracts strongly captures the narratives of the participants:

I was thinking in my head I still had the power and I am in control of this by trying to show her I am not interested so that would make her more interested … so in my head I am probably thinking yeah I will show her … it will probably make her work as hard to get me back …I was still trying to put a brave face on that I wasn’t interested and I didn’t care so she will want me more than I want her … its making me feel good isn’t it because I am thinking I have got the control (P5, 478–495).

As much as I don’t want to say that, I don’t like it, erm its more of a power thing …it becomes a game and it becomes quite manipulative at times where you are manipulating things to get what you want to get that acceptance … as much as I don’t like to say (…) and I don’t think it’s even fully right, its controlling … (P1, 576–585).

Within the aforementioned extracts, a striking aspect is how participants articulate a narrative of power and control as explanations for their behavior. A striking aspect is that the goal is to reconcile, but the way in which reconciliation is achieved is through bringing an element of the thrill of the chase and gameplaying. From a hermeneutic level there is a sense that the ultimate goal is to elevate the sense of self and heighten positive feelings to mask insecurities. There is a strong compulsion to win to avoid humiliation, ridicule, or perceived loss of power and the need to affirm the dominant winner’s position. Central to this theme is how participants responded to legal sanctions and the gameplaying that manifests with professionals when legal sanctions are imposed. Participants described continuing their behaviors despite continued police warnings and sanctions from the courts. From a hermeneutic level, a willingness to breach orders is reflective of the view that what they are gaining from the stalking behavior is more important to them than the personal costs. This following extract encapsulates the narratives of participants:

The police said … you need to stay away from the area, but I stood there texting her going oh yeah whatever, and the police woman said what are you doing, I said texting her, she said haven’t you been listening? … I said listening, listening to what … she said you have to stop a minute sarg he has already text her, he said have you text her I said yeah … he said you wasn’t listening, I said yeah, he said what did I say, I said stay away from X … and they went so why have you text her? … So, I started writing letters and started posting them and I got done for breach of bail three times erm I was told you got told not to text her or ring her. I said I didn’t I said I sent letters. I said you said don’t ring or text which I didn’t, so I sent letters instead cos you didn’t say that and she was like oh are you trying to be clever, and I was well you didn’t say I couldn’t write her letters, you said don’t ring her or text her so I didn’t … she said alright don’t ring her, don’t text and don’t send letters but they her birthday came up so I sent 50 cards and sent her some flowers. so, the police came again for breach of bail cos I had sent the cards …(P2, 71–85).

### Superordinate Theme 5: “Severed Connections, Changing the Gameplay”

This salient theme represents the emotional tipping point and the subsequent pathway to seek to reconcile or destroy the victim in the later stages of relationship dissolution. The theme takes two interlinked directions. Firstly, the theme reflects the emotional response to facing rejection and unrequited love. Secondly, it conceptualizes how when faced with the reality of rejection, participants experienced an emotional tipping point and extreme behavioral response, paving the way for violence. This pathway starts with attempts to reconcile by making contact. When this fails, this escalates to gameplaying and sexual or physical violence. A striking aspect of this theme was the ambivalence and paradox within participants’ accounts and how focus shifted from pursuit to revenge and the desire to reconcile. Whilst there were some variations across participants in how this played out in the offense pathway, there were striking similarities in how this manifested in the subordinate themes. Whilst this theme was pertinent to the pathway to IPS from participants in this study, with participants engaging in significant violence towards the victim, it should be noted this superordinate theme may not be apparent in IPS perpetrators whose stalking does not evolve in a high level of violence.

#### Spiraling emotions: “In one of those snowdomes going around in circles.”

This theme conceptualizes the rejected and emotional self, encapsulating how participants experienced struggling with rejection. This extreme emotional response was the catalyst for violence perpetration in the later stages of the offense pathway. Reflecting on the pathway to their stalking behavior, a central feature resonating throughout participants’ narratives was the feeling of rejection, abandonment, and loneliness. This extreme emotional response was the catalyst for violence perpetration. The following extracts exemplify the experiences of participants when faced with rejection seeing the emergence of the emotional and questioning self:

She wasn’t there, desertion at the worst possible time (P4, 290–291). I was in one of those snowdomes going round and round and round in circles … but I am getting nowhere … A roller coaster, life is a roller coaster … especially my life, you wouldn’t want to be in my shoes (P6, 310-313). I thought nobody wants me sod it (P6, 206). Obviously, it hurt me … I was thinking what the fuck … am I not good enough for you (P5, 394). I didn’t want to let her go, there was just something in my heart telling me I don’t want her to leave me and I think it was I don’t want to be on my own (P5, 261–262).

One notable feature underpinning participants’ narratives was the sense of emotional pain recounting these experiences. Through the collective narratives, participants spoke of spiraling emotions and how feelings of love rapidly turned to hate:

The feelings I had was painful, I felt useless … it was hurting bad, and it was all confusion cos it was playing with my head … (P5, 200–220). I hated her … I was thinking why has she done this to me … (P5, 407–409). I felt I hated her, and that’s how I felt (P1, 222). I think I kind of hated her (P2, 56).

There is a sense that through participants’ behavior the aim was to reconnect or stabilize the equilibrium in order to soothe the emotional pain of rejection. It is at this point that participants expressed believing the relationship could be saved, despite their behavior. This is exemplified in the following extracts:

I still suffered that feeling that I could somehow sort of reestablish it so for me one of the issues that I suppose is going on is that it is understanding when end means end … (P3, 768–769). At the time I honestly believed that we could sort it out. I was naïve … I couldn’t imagine life without her (P4,120). Me virtually telling myself regardless of what she said to me is we will get back together … it was me having such strong thoughts and beliefs that it sort of sent me into the devil’s care (P1, 561–563).

#### Restoring pride and elevating the self: “A red rag to a bull.”

This theme represents the pathway from pursuit to revenge and the tipping point to violence and extreme levels of sadistic stalking in the later stages of pathway to IPS. This theme stems from how participants described responding to rejection coupled with feelings of betrayal or humiliation, leaving the true self exposed. Throughout participants’ narratives was a sense of retribution for perceived wrongdoing driven by public exposure, ostracism, and humiliation. With participants shifting emotions, and when love turns to hate, any connection was perceived to be better than none. This fueled a desire to repay harm with harm. This is exemplified in the following extracts:

I threatened her verbally on the phone once (you will get what you deserve) …. But that wasn’t a threat of violence … I had sat back and laid back and taken all of this … the theft, the lies, the denial of closure, the lack of respect for what I had done for her family, erm the adultery (P4, 321). I am not going to lie … I wanted to hurt her from all what she had done … (P4, 397–400). My original one was just to humiliate her … because that is what she had done to me erm it was that simple.” (P4. 252–255). I stopped her becoming a X, I wrote to the X … hands up I wrote a letter, I didn’t hide anything it was upfront, I was actually acting in public interest, there was no way on earth this woman should be allowed in any way shape or form to become a X … I sent erm a letter or an email to her and some of her friends erm outlining who she was, what she was, what she had done (P4, 256–264).

She made a formal complaint to the police and I was charged with breaching the peace. That was just like a red rag to a bull … I was then referred down to the inpatient service facility … I just sat there and stewed … I just thought I need to get her back and try and sort things out … (P3, 585–597)….So basically, I had backed myself into a corner …I knew the outcomes erm as soon as the police became involved with the second victim, I knew I had ended up walking myself into a trap and I had nowhere to go (P3, 877–888).

It was the humiliation and definitely the stain on my character … That would turn me probably to extreme violence … you should have told me that you erm this is not the relationship you wanted to be in instead of lying and allowing it to continue …I came out of prison probably the angriest man you have ever met because I thought okay you put me in prison you I am going to have to do something here I am going to have to show you I am not going to take that shit … I knew she was going to get hurt (P7, 396–491).

Whilst the tipping point is rejection, there is a sense that it goes deeper than this. The tipping point is exposure and a total perception of ostracism and feeling that the whole world is looking in. From a hermeneutic level, there is a sense that this is viewed as the ultimate betrayal, which is the catalyst for the process of dehumanization in order to overcome shame. Within the collective narratives, there is a sense of willful dominance and it is becoming a conquest, a game where participants’ view the self as winning or losing.

## Discussion

The study explores the pathway to IPS through the eyes of the perpetrator using a phenomenological approach to capture the “voice,” experiences, and cognitions of men who have engaged in IPS. Consideration is now given to synthesizing the findings. Each superordinate theme is discussed in turn with reference to the wider psychological literature and theoretical models of stalking. Implications for theory and forensic practice are discussed.

## Contributions of the Study: Implications for Theory and Forensic Practice

### Superordinate Theme 1: The Quest for Attention and Affection Creating Connection

The findings can be placed in the context of attachment (Patton et al., 2010), psychodynamic (Meloy, 1998), evolutionary/sociobiological (Duntley & Buss, 2012), and relational goal pursuit (Cupach & Spitzberg, 2014) models of stalking. From a hermeneutic level, participants’ accounts are explained by attachment deficits and a desire for closeness, and hypersensitivity to rejection, with relationship styles which appear to be based on extreme sexual attraction, obsessive thinking, possessiveness, and dependency. There is a sense that participants hold underlying social scripts that in the face of rejection if you persist, then love will conquer all. Placed in the context of RGP (Cupach & Spitzberg, 2014), from a hermeneutic level, the findings can be explained by the strong desire to achieve the goal of attaining the relationship and how this brings higher order goals of self-worth and a perceived sense of happiness. Fisher’s (1998) neurobiological model of love and the work of Meloy and Fisher (2005) provide an important context for this superordinate theme. This model explains stalking from a neurobiological perspective on the psychology of “romantic love,” postulating that the attachment system becomes activated in response to various stages of rejection in the stalking pathway. The initial lust and attraction phase are characterized by desire and craving the other, driven by the desire to achieve sexual gratification. In response to rejection, Fisher (2004) refers to the Romeo and Juliet effect or “frustration–attraction,” leading to emotional dependence, obsessive thinking, and intense sexual desire. For the participants in this study, the lust and attraction phase are captured in the narratives of participants. Participants appear goal orientated and strongly motivated to connect in the early stages of the relationship. Significantly, participants in this study appear stuck in the lust and attraction phase of a relationship.

### Superordinate Theme 2: Conflicted Identity and Extremes of Self

The findings underpinning this superordinate theme can be explained to some extent by RGP (Cupach & Spitzberg, 2014) and coercive control theory (Dutton & Goodman, 2005; Stark, 2009). From a hermeneutic level, it is interpreted that when life was going well, participants held the perception of a high level of control in their lives. In response to life problems and rejection, they attempt to regain control over various domains of their lives (i.e., work, relationships, and status), impacting on the sense of self and attempts to regain control. In response to control deficits, the participants in this study can be seen to exercise control, culminating in attempts to maintain dominance and an act of IPV. The experience of loss appears to be central to their experiences, mirroring the view of Mullen et al. (1999) that loss is often combined with frustration, jealousy, anger, vindictiveness, and sadness. The findings can be placed into context of the shame–aggression perspective (Velotti et al., 2014). That is, participants experienced overwhelming negative emotions of embarrassment and humiliation, underpinning shame, leading to devaluation of the self. As a protective strategy, participants appear to project self-hatred onto victims, believing the victim is responsible for their suffering. Feelings of shame emerge in response to perceptions of social exclusion (Dickerson et al., 2009), culminating in emotional discomfort and anger which motivate aggression (Davey et al., 2005). Consequently, the pathway to IPS can be explained by a psychobiological chain linking shame to anger and aggression.

#### Superordinate Theme 3: My Life, a Film Set

The cognitive characteristics of rumination and obsessive thinking can be explained to some extent by RGP (Cupach & Spitzberg, 2014). Participants’ experiences of rejection and the recognition of not fulfilling the primary goal of the relationship with the victim, triggered rumination that their goal remained unsatisfied, enhancing the belief that achieving intimacy with the victim will increase self-worth (Cupach & Spitzberg, 2014). From a hermeneutic level, there is a sense of participants being a trickster character, in that they were the ones mixing up the messages or even actively choosing to ignore the signs and reframe them to suit their needs. This finding supports Cupach et al. (2000), who suggest stalking perpetrators misinterpret rejecting behaviors as encouragement by the victim and fail to understand the negative impact of their behavior.

#### Superordinate Theme 4: Gameplaying: “One Step Ahead”

It is argued that the fixated and obsessive nature of coercive control parallels the fixated and obsessive nature of stalking (Monckton-Smith et al., 2017). From the perspective of coercive control theory, Stark (2009) would suggest participants’ surveillance and monitoring of their partner, whilst the relationship was intact, was part of a pattern of coercive control. Whilst participants described feeling their stalking behavior was driven to some extent by control, they recognized that there was an additional layer to this explanation, which was much deeper than control and was about winning. Consequently, there are gaps in how coercive control theory seeks to explain the pathway to IPS. The overall findings support the view that whilst stalking behavior and coercive control are interlinked, for the participants in this study, the findings indicate that stalking behavior differs from coercive control. Alternatively, the psychology of motivation and the desire to win provides an important context for the findings underpinning this superordinate theme (Breiter et al., 2001). This theoretical explanation suggests that the dopaminergic reward system is activated by a motivation to win.

#### Superordinate Theme 5: “Severed Connections, Changing the Gameplay”

The findings underpinning this superordinate theme can be explained by a psychodynamic perspective (Meloy, 1998) and theories of aggression and violence, specifically the role of self-conscious emotions (i.e., embarrassment, shame, and humiliation) which are deemed significant drivers for violence (Walker & Knauer, 2011). Anger, hate, rage, and resentment were noteworthy emotions in the later stages of IPS for all participants. A loss of status and threats to self-esteem were central to the pathway to IPS, and appeared to lead to cognitions underpinned by a desire for control and need to win which culminated in acts of violence. Central to participants’ experiences was the sense that events in the pathway to stalking violence had initiated a catathymic reaction, with revenge providing relief from emotional and psychological turmoil (Schlesinger, 2007). It is noteworthy that social rejection and exposure of the true self was the catalyst for violence perpetration. In all cases, this created a sense of internal conflict, coupled with an intense negative affect of anger, hate, resentment and fear (of failure) which was inflicted upon the victim.

#### Contributions of the Study: Implications for Forensic Practice

The findings have strong implications across a range of criminal justice system settings, specifically for international policymakers, and informing guidance on intervention approaches for IPS perpetrators. Firstly, the findings further illuminate the debate regarding the conceptualization of IPS. Central to the experiences of participants was a prior history of psychological violence, and in some cases physical violence, within the relationship history with the victim. This finding suggests that both psychological and physical violence played a role in the pathway to IPS. Whilst all participants described engaging in stalking behavior during the relationship and following the dissolution of the relationship, there were differences in how this played out in the pathway to IPS. That is, some described using surveillance tactics in the attraction stage of relationship development, tactics which they continued to use throughout the relationship, and following the dissolution of the relationship, whereas others engaged in stalking behaviour during the relationship and post relationship. The relationship histories and experiences of participants indicate there are likely subtypes of IPS perpetrators. To this end, the findings support the existing literature, indicating a connection between IPV and stalking behavior. Unpacking this further, from the experiences of participants in this study whilst the findings suggest IPS may be a continuation of prior IPV, there is also another layer, suggesting that whilst IPS and stalking behavior are related patterns of behavior with some shared psychological underpinnings, there are some distinct behaviors playing a role in the pathway to IPS violence.

Secondly, the use of IPA has provided insight into the cognitive characteristics of this group, representing what is known to be the first qualitative attempt to form a picture of the cognitions of IPS perpetrators. The cognitive characteristics of IPS perpetrators can be explained to some extent by the IPV implicit theory (IT) literature (Gilchrist, 2009; Weldon & Gilchrist, 2012). Participants’ accounts suggest there are some similarities in the cognitive characteristics of IPS perpetrators and men who commit IPV offenses, whereas some are specific to IPS perpetrators. “ITs” of “Womens’ role in violence,” “Women as objects,” and “Entitlement” as central to the cognitions of IPS perpetrators underpinned participants’ cognitions in Superordinate theme 1: “The quest for attention and affection creating connection.” The study identified a new “IT” of “Obsession and fantasy” specific to IPS perpetrators. “Superordinate theme 2: Conflicted identify and extremes of self” identified “ITs” of “Real man,” “Out of control,” “Uncontrollability,” and “External factors responsible” underpinning the cognitive characteristics of participants. There was the sense that stalking behavior was in some way out of participants’ control, in that external factors (i.e., loneliness, substances, other women or illness) were responsible and played a central role in the pathway to IPS. In the lead up and during the stalking campaign participants perceived they were losing control of some element of their life and relationship. “Superordinate theme 3: My life, a film set” identified the “ITs” of “Women’ role in violence,” “Rejection/abandonment” and “Entitlement.” Central to participants’ experiences was ambivalence towards the victim and relationship, and the push/pull factors of the relationship. “Superordinate theme 4: Gameplaying ‘one step ahead’” identified the “ITs” of “Win or lose” in the cognitions of IPS perpetrators. Specific to IPS perpetrators is the “IT” of “Knowledge is power.” This explains how individuals see knowledge as a powerful tool to navigate situations and enable them to monitor and stay one step ahead. “Superordinate theme 5: Severed connections, changing the gameplay” identified the “ITs” of “Win or lose” and “Grievance/revenge” as present in the cognitions of IPS perpetrators. For participants in this study there was a collective sense that violence is an acceptable response when slighted and rejected. These “ITs” have the potential to be transformed into treatment needs (Dempsey & Day, 2010) and inform the design of interventions. The findings suggest that IPS may have a greater level of criminogenic need compared to IPV perpetrators. Furthermore, the overall findings illustrate that IPS perpetrators are not a homogenous group and as such are not compatible with a “one-size-fits-all” approach to intervention.

Thirdly, issues specific to the presentation of IPS perpetrators warrant consideration for how professionals interact and engage with this group within the criminal justice system.

The findings illustrate how “gameplaying” and a “desire to win” were central in the pathway to IPS, which subsequently played out in response to legal sanctions. Consideration should be given to this presentation throughout contact with the criminal justice system. Initially from the perspective of the police and courts, and subsequent supervision measures in a custodial and community setting, through to how this may manifest in approaches to clinical management and the potential for these to act as barriers to the development of therapeutic alliance and engagement in intervention.

## Limitations of the Current Study and Directions for Future Research

Despite the theoretical and applied contributions of this study, it is not without limitations, and conclusions must be drawn with caution. The sample was purposively selected from several prison settings. It is acknowledged the sample represents perpetrators with a higher level of risk whose behavior had escalated to stalking violence. Consequently, the generalizability of the study is limited by the context in which the study took place. It cannot be concluded whether the pathway to IPS would be similar or different to perpetrators who did not escalate to violence. Indeed, it cannot be concluded whether female perpetrators or those from diverse cultural backgrounds would construct meanings of their experiences in a similar way to participants in this study. Future research should build on and replicate a similar research design with a sample of IPS perpetrators who are serving a community sentence for a stalking conviction. This would unpack whether the themes identified in this study are specific to IPS perpetrators. Given the lack of studies exploring desistance and the role of protective factors, qualitative studies focusing on this area warrant exploration.

## Conclusion

The study provides a unique contribution to the stalking literature by presenting the first known phenomenological approach to develop a rich understanding of the profile of men who have engaged in IPS. Participants in this study described engaging in stalking-related behaviors during the relationship and following the breakdown of the relationship in an attempt to reconcile or seek revenge, with evidence of psychological and physical violence during the relationship. The findings indicate that the pathway to IPS stalking and stalking violence is complex and underpinned by a multitude of interacting cognitions, emotions, and situational factors. Whilst there are similarities in the cognitive characteristics of IPS and IPV perpetrators, there are several critical cognitive characteristics that underpin IPS. The research has the potential to inform intervention approaches and has strong application to forensic practice.

## Author Biographies

**Caroline Flowers** Doctor of Psychology in Forensic Psychology. She is a lecturer in Forensic Psychology at Nottingham Trent University. In 2019 she completed a doctorate in forensic psychology at Nottingham Trent University in the area of intimate partner stalking. Her current research interests include intimate partner violence, stalking behaviour, and approaches to intervention for intimate partner stalking perpetrators.

**Belinda Winder,** PhD, is a professor of Forensic Psychology. She is research director of the Centre for Crime, Offending, Prevention & Engagement (COPE) in the School of Social Sciences at Nottingham Trent University, and part of the Sexual Offences, Crime and Misconduct Research Unit (SOCAMRU) in the Department of Psychology at Nottingham Trent University. The main focus of her work is applied research in the area of sexual offending and sexual crime, but she also has an interest working with people with lived experience, desistance research, and research that contributes to facilitating those with convictions to lead a purposeful life post-conviction.

**Karen Slade,** PsychD, is an associate professor of applied forensic psychology at Nottingham Trent University and a registered forensic psychologist. Her research and practice focus on the areas of self-harm, suicide, and violence in forensic populations, the impact on prison staff of working in difficult environments, mental health services for offenders, and organizational approaches to suicide and violence prevention.
